# Unravelling the many facets of human cooperation in an experimental study

**DOI:** 10.1038/s41598-023-46944-w

**Published:** 2023-11-10

**Authors:** Victoria V. Rostovtseva, Mikael Puurtinen, Emiliano Méndez Salinas, Ralf F. A. Cox, Antonius G. G. Groothuis, Marina L. Butovskaya, Franz J. Weissing

**Affiliations:** 1 Groningen Institute for Evolutionary Life Sciences, University of Groningen, Nijenborgh 7, 9747 AG Groningen, The Netherlands; 2 Institute of Ethnology and Anthropology, Russian Academy of Sciences, Leninsky Prospect 32a, Moscow, Russia 119334; 3 Department of Biological and Environmental Science, University of Jyväskylä, Survontie 9 C, 40014 Jyväskylä, Finland; 4 Faculty of Behavioural and Social Sciences, Developmental Psychology, University of Groningen, Grote Kruisstraat 2/1, 9712 TS Groningen, The Netherlands; 5 Netherlands Institute for Advanced Study in the Humanities and Social Sciences, Korte Spinhuissteeg 3, 1012 CG Amsterdam, The Netherlands

**Keywords:** Human behaviour, Social anthropology

## Abstract

Humans readily cooperate, even with strangers and without prospects of reciprocation. Despite thousands of studies, this finding is not well understood. Most studies focussed on a single aspect of cooperation and were conducted under anonymous conditions. However, cooperation is a multi-faceted phenomenon, involving generosity, readiness to share, fairness, trust, trustworthiness, and willingness to take cooperative risks. Here, we report findings of an experiment where subjects had to make decisions in ten situations representing different aspects of cooperation, both under anonymous and ‘personalised’ conditions. In an anonymous setting, we found considerable individual variation in each decision situation, while individuals were consistent both within and across situations. Prosocial tendencies such as generosity, trust, and trustworthiness were positively correlated, constituting a ‘cooperativeness syndrome’, but the tendency to punish non-cooperative individuals is not part of this syndrome. In a personalised setting, information on the appearance of the interaction partner systematically affected cooperation-related behaviour. Subjects were more cooperative toward interaction partners whose facial photographs were judged ‘generous’, ‘trustworthy’, ‘not greedy’, ‘happy’, ‘attractive’, and ‘not angry’ by a separate panel. However, individuals eliciting more cooperation were not more cooperative themselves in our experiment. Our study shows that a multi-faceted approach can reveal general behavioural tendencies underlying cooperation, but it also uncovers new puzzling features of human cooperation.

## Introduction

Cooperation has attracted scientific attention for more than a century^1–5^. Cooperation in our own species is of particular interest: humans cooperate in a diversity of contexts, not only with relatives and friends but also with unrelated individuals and even strangers, and in the absence of any opportunity for reciprocal interactions in the future^6–10^. Human cooperation is a multifaceted phenomenon, which involves a large number of different behavioural components and social qualities, such as generosity, readiness to share with others, fairness, trust and trustworthiness, and willingness to take cooperative risks. In addition, an important constituent of cooperation is the coordination of actions with those of others.

The results of several studies show that against the background of the general cooperative nature of humans, there are considerable individual differences in prosocial predispositions, which, to a certain extent, appear to be temporally and contextually stable^11–13^. At the same time, only a few individuals are unconditionally cooperative/selfish^14–16^. Unconditional behaviour has strategic disadvantages, as pure altruists can be exploited by selfish individuals, while unconditionally uncooperative partners may be avoided by others. Therefore, both individual consistency and flexibility are involved in cooperative behaviour at the same time^17–19^. Behavioural flexibility involves responsiveness to a particular social environment and is only profitable if there are predictable (and hence consistent) individual differences in behaviour. Therefore, consistency, flexibility, and predictability may be viewed as three fundamental and interrelated aspects of human cooperation.

To better understand human cooperativeness, we designed an experiment that allows us to disentangle the different components of cooperativeness and explore their interplay. Our study has three key ingredients. First, we assessed various prosocial behavioural qualities (generosity, trust, trustworthiness, fairness, risk-taking, free-riding, work share, coordination, demand, and punishment) in different experimental contexts (decision situations). This allows us to quantify the degree of individual variation in prosocial qualities, as well as the sign and degree of correlation between these behavioural traits. Second, the individuals of our study were tested three times in each context, with three different interaction partners: they first had to make a decision in an anonymous setting (without any information about their interaction partner), and subsequently they had to make two similar kinds of decision in a ‘personalised’ setting, after seeing a silent facial video of their interaction partner. This allows us to investigate whether and how individuals make their decisions dependent on their interaction partner’s appearance, and to what extent different individuals respond similarly to the same interaction partner. Third, we study whether specific aspects of the visual appearance of the individuals shown in the videos can explain the individuals’ effect on the decisions of their interaction partners and whether these aspects are correlated with the individuals’ own decisions in the experiment. This allows us to find out whether partners eliciting prosocial behaviour are prosocial themselves.

Quite a number of studies focusing on cooperation and free-riding in the context of the Prisoner’s Dilemma Game and the Public Goods Game have previously reported consistent individual variation in cooperation tendency^14–16,20^. However, there is more to cooperation than the problem of free-riding. Indeed, free-riding plays a minor role in other aspects of cooperation (and the games designed to study these aspects). We therefore address the question of whether consistent individual variation in prosocial tendencies is also observed in these other games, and whether prosocial dispositions are correlated across games. Five earlier studies tackled similar research questions. Four of these had a different focus than the present study: social value orientation^12^, reciprocity^21^, age effects^22^, and consistent variation in human social learning strategies^23^. The fifth study^13^ may be viewed as the starting point of our current study. Peysakhovich and colleagues^13^ conducted a large-scale online study where individual prosocial dispositions were investigated in eight economic games related to cooperation. The first, anonymous-interaction part of our experiment has a similar set-up as the study in Peysakhovich et al.^13^, but it differs in various respects from it: interactions took place on-site, the games were presented with a very different narrative, and the incentives were much higher (more than 20-fold). Therefore, the first part of our experiment sheds light on the repeatability and robustness of the main finding of Peysakhovich and colleagues, the existence and structure of a “cooperativeness syndrome.”

The second part of our experiment goes an important step further by personalising the interactions, thus allowing us to explore the effects of the interaction partner’s appearance on prosocial dispositions. Responsiveness to the social environment constitutes an important part of human behavioural plasticity. The appearance of one’s interaction partners can be an important cue for this environment, especially under conditions when a reputation-based way of building cooperation^24–26^ is not available (e.g. in large groups, or in a new social environment). A number of studies provide evidence for the ability of humans to predict the social behaviour of others by only visual information^27–31^. Therefore, to test whether behavioural reactions induced by a certain appearance in our study were based on specific visual traits, the faces of our subjects were judged by a separate assessment panel. The panel members had to score same-sex neutral facial images regarding various attributes on a five-point scale. The list of attributes covered a range of perceived traits, including pro- or antisocial qualities (trustworthiness, generosity, greediness), emotional dimensions (happiness, angriness), and attractiveness. This allowed us to investigate whether these attributes (1) had an effect on the interaction partners’ decisions in the personalised setting and (2) were correlated with the individuals’ own behaviour.

## Results

Each individual in our study was involved in 10 different decision situations, and each of these situations was experienced three times: once in an anonymous setting (without any information about the interaction partner), and twice under personalised conditions (after seeing a short silent video of the interaction partner). To avoid possible interference between prosocial and sexual attitudes, we focused on same-sex interactions. The decision situations were designed to roughly correspond to well-known cooperation games^32,33^ but were presented in an everyday context. Brief descriptions for each decision situation are given in Table 1. More details on the rules, strategies, and payoffs can be found in the “Methods” section (Table 2) and in our extensive study protocol^34^.

**Table 1:** Brief description of the ten decision situations in our experiment.

Aspect	Game	Interaction context
Generosity	Dictator Game (DG)	The subject has to decide how much money to donate to an unknown interaction partner
Trust	Trust Game (TG1)	The subject has to decide whether to entrust their goods to an unknown market seller, who promises to sell the goods with a profit and share the profit with the trustor
Trustworthiness	Trust Game (TG2)	The subject is a market seller, who can either return to the trustor and share (part of) the profit or keep all the profit by themselves
Fairness	Ultimatum Game (UG1)	The subject finds a winning lottery ticket and offers a fraction of the price to an unknown bystander. If the bystander does not like the offer, they can carry the ticket to a lost-and-found office and both end up with nothing
Demand	Ultimatum Game (UG2)	The subject is the bystander, who decides on the minimal acceptable offer from the finder of the winning ticket in order to share in the prize
Risk-taking	Stag Hunt Game (SH)	The subjects are sellers of peaches, for which there is a fixed demand in the buyer community. The peaches can be sold for a high or a low price. If both sellers decide on the high price, each sells with maximum profit. The risk is that the other seller undercuts the price, thus selling all peaches, and leaving the expensive seller empty-handed
Punishment	Punishment Game (PG)	The subjects are asked to imagine that the above risk-taking game was played a second time. They can punish bad behaviour of the competitor by setting an even lower price for peaches and decide when to do so: (a) never; (b) if their price was undercut by the other seller; (c) always
Free-riding	Prisoner’s Dilemma (PD)	The subjects are two sportsmen who are ahead of the crowd in a cycling race. Each has to decide whether to take the leading position exposed to the wind (cooperate) or to go under the partner’s shelter (defect). Taking turns in the lead increases the likelihood that one of the two will win the race, but the one most often exposed to the wind has the smaller likelihood of being the winner
Work share	Snowdrift Game (SD)	The subjects are two students who have to write a common report together. Each student has to decide how many points (0 to 100) to invest in report writing. Both get the same credits for the report, but only if the report is completed (the sum of invested points exceeds 100)
Coordination	Coordination Game (CG)	The subjects are two friends who want to meet each other but have no way to coordinate this. Each has to decide on one of two possible meeting places: the city library or the university library. Both know that the favoured location of subject A is the city library, while the favoured location of subject B is the university library

See Table 2 for detailed payoffs, and the Study Protocol^34^ for how the game situations were presented to the participants in the experimental instructions.

**Table 2:** Rules and payoffs of the economic games used in our study.

Aspect	Game	Game rules
Generosity	Dictator Game (DG)	One-sided decision to donate 0 ≤ *x* ≤ 100 points to the interaction partner, keeping 100-*x* points for themselves
Trust	Trust Game (TG1)	The trustor decides whether to entrust 50 points to the trustee. If entrusted, the number of points is tripled, and the trustee decides how many points (up to 100) to return to the trustor. Unreturned points are kept by the trustee
Trustworthiness	Trust Game (TG2)	
Fairness	Ultimatum Game (UG1)	Player 1 makes proposal on the allocation of 100 points between self and Player 2. Player 2 may either accept the proposal (and the proposal will be effectuated) or reject the proposal (and both players receive nothing)
Demand	Ultimatum Game (UG2)	
Risk-taking	Stag Hunt Game (SH)	Decision to cooperate (C) or to defect (D). Payoff matrix (payoffs of the row player):
Punishment	Punishment Game (PG)	By spending 30 points, a player can take away 100 points from the other player. The player has to decide when to do so: (a) never; (b) if partner chose D in the previous Stag Hunt Game; (c) always
Free-riding	Prisoner’s Dilemma (PD)	Decision to cooperate (C) or to defect (D). Payoff matrix (payoffs of the row player):
Work share	Snowdrift Game (SD)	Each player decides how many points (0–100) to invest in a common pool with the other player; points not invested are kept. If the pool contains 100 points or more, each player gets 160 points, in addition to the points not invested
Coordination	Coordination Game (CG)	Each of two players has to decide between two options A and B. Player 1 prefers option A, and player 2 prefers option B. If both players choose A, player 1 gets 100 points, and player 2 gets 30 points. If both players choose B, player 1 gets 30 points, and player 2 gets 100 points. If they choose different options, both get nothing

### Variation in prosocial tendencies

**Figure 1:**
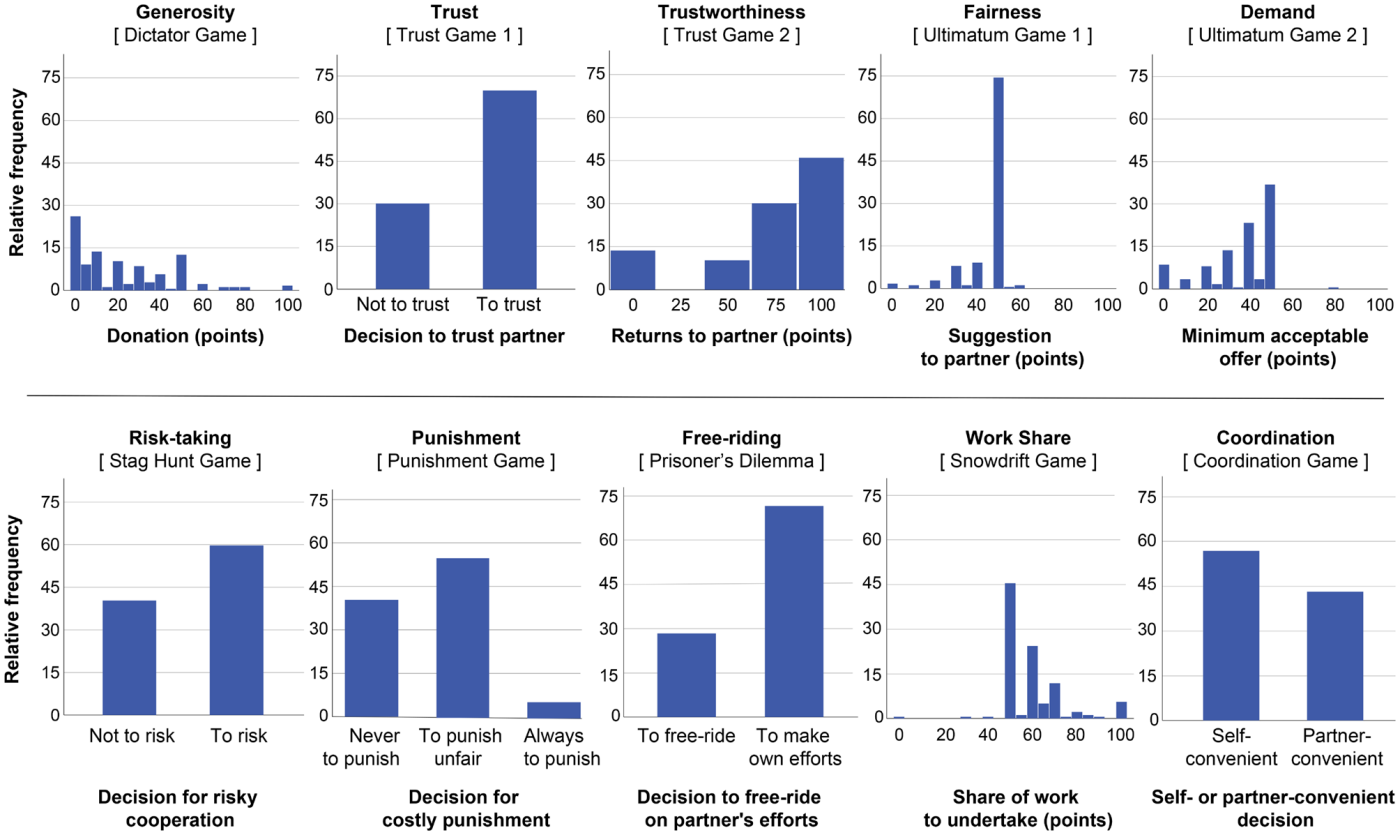
Individual variation in prosocial decisions. Relative frequency distribution of individual decisions (N = 176 per plot; anonymous conditions) in the ten experimental situations.

Figure 1 shows the relative frequency distributions of the decisions taken under anonymous conditions in the ten experimental situations. In all situations, there was considerable variation in behaviour. In the Punishment Game, only a few participants chose to punish always. For simplicity, we excluded these cases from further analysis of the Punishment Game and assigned a binary output to this game: [0] never punish or [1] punish if the interaction partner behaved unfairly in the previous game.

### Correlations of prosocial tendencies across games

Next, we analysed whether, and to what extent, the subjects in our experiment were consistent in their decisions across games. To this end, we quantified, in the anonymous setting, the association between prosocial decisions in the ten experimental situations. As a statistical tool, we used categorical Principal Component Analysis (catPCA), which allows for combining differently scaled variables (nominal, ordinal and continuous).

**Figure 2:**
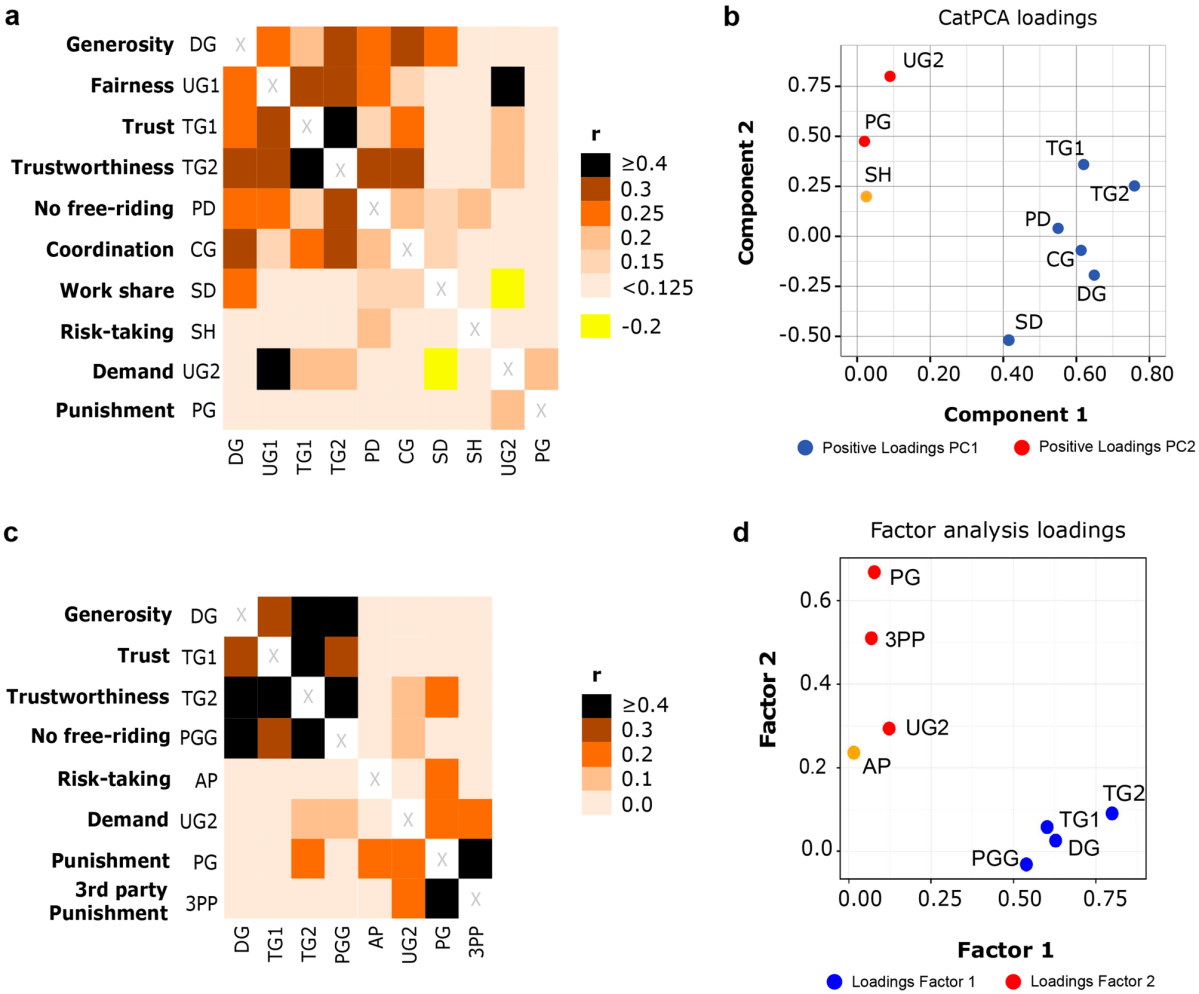
Associations between decisions in different experimental situations in two studies. Comparison of our results (**a**, **b**, anonymous interactions) with those of Peysakhovich et al. (2014) (**c**, **d**). (**a**) Correlation matrix for the decisions in the ten situations in our experiment: Dictator Game (DG), Ultimatum Game (UG1, UG2), Trust Game (TG1, TG2), Prisoner’s Dilemma (PD), Coordination Game (CG), Snowdrift Game (SD), Stag Hunt Game (SH), Punishment Game (PG). Heatmap based on Pearson’s correlation coefficients (transformed through catPCA, N = 168). (**b**) Principal component loadings of the decisions from the catPCA of our experiment (N = 168). Variance explained: Comp 1 = 25.2%; Comp 2 = 15.4%; Total = 40.6%. The risk-taking game (SH) was not significantly loaded on any of the components, while work share (SD) was loaded on both components. (**c**) Correlation matrix for the decisions in the games in the study of Peysakhovich et al.^13^: Dictator Game (DG), Ultimatum Game (UG2), Trust Game (TG1, TG2), Public Goods Game (PGG), All-pay Auction (AP), 2nd Party Punishment Game (PG); 3^rd^ Party Punishment (3PP). The games were relabelled, and their order was rearranged to facilitate comparison with the correlation matrix of our study in (**a**). (**d**) Factor analysis loadings from the experiment of Peysakhovich et al.^13^. Total variance explained: 79%. The risk-taking game (AP) was not significantly loaded on any of the factors. The difference in axes labelling in (**b**) and (**d**) corresponds to the difference in the methods used in our study (categorical Principal Component Analysis) and the study of Peysakhovich et al. (factor analysis). See Supplementary Table S2 for more details on the study of Peysakhovich and colleagues and our relabeling of their types of decisions to facilitate comparison with our study.

Figure 2a shows a heatmap of the correlation matrix (numerical values are given in Supplementary Table S1). Any correlation coefficient with absolute value above 0.15 is statistically significant at the 0.05 level. Most correlations between the prosocial decisions—generosity (DG), fairness (UG1), trust (TG1), trustworthiness (TG2), workshare (SD), absence of free-riding (PD), and coordination (CG)—were positive and significant, indicating a “cooperativeness syndrome.” In other words, individuals that take a prosocial (resp. antisocial) decision in one of the corresponding seven experimental situations tend to choose a prosocial (resp. antisocial) option in the other situations as well. Costly punishment (PG), which is considered prosocial in cooperation theory^6^, was not positively correlated with prosocial behaviour in the seven decision situations above. The same holds for demand (UG2), which may also be viewed as a form of punishment. In fact, a high demand level is positively correlated with the tendency to punish (Supplementary Table S1). Last but not least, risk-taking (SH) is also not part of the cooperativeness syndrome. It is only positively correlated with cooperation in the Prisoner’s Dilemma, which makes sense, as cooperation in the PD faces the high risk of being exploited by a free-rider. Figure 2c shows the heatmap of the corresponding correlation matrix in the earlier study of Peysakhovich and colleagues^13^. For ease of comparison, we rearranged the order of games in Peysakhovich et al.^13^ and labelled the various types of decisions as in our study. The comparison of Fig. 2a and c reveals that the correlation structure obtained in both studies largely agrees, despite the fact that the two studies differed in various respects (slightly different games, different narratives, different payoffs, different experimental settings; see Supplementary Table S2 for details).

Figure 2b shows the results of the categorial Principal Component Analysis (catPCA) for the anonymous interactions of our study, while Fig. 2d reproduces the factor analysis in Peysakhovich et al.^13^. As Peysakhovich et al.^13^ did not include an aspect corresponding to the “fairness” part of the Ultimatum Game (UG1), we excluded this part from our analysis. The catPCA of the experimental results of our study reveals two main components, which explain about 40% of the total variance. The prosocial decisions regarding generosity (DG), trust (TG1), trustworthiness (TG2), work share (SD), absence of free-riding (PD), and coordination (CG) loaded positively on Component 1, whereas punishment (PG) and demand (UG2) loaded positively on Component 2 (Fig. 2b). This corresponds very well with the results of Peysakhovich and colleagues (Fig. 2d). Interestingly, the “risk-taking” decisions (SH in our experiment, and AP [All-pay Auction] in Peysakhovich et al.^13^) were not loaded on any of the components in both studies.

Summarising, Fig. 2 suggests that the findings reported here and in Peysakhovich et al.^13^ are replicable and quite robust. However, we would like to conclude the section on anonymous interactions with a caveat. If we re-run the catPCA with the inclusion of the “fairness” part of the Ultimatum Game (UG1), Component 2 differs considerably from that in Fig. 2b (see Supplementary Figure S1). This is an illustration of the well-known fact that Principal Component Analyses are sensitive to the number of data dimensions.

### Effect of partner’s appearance on prosocial behaviour

One of the goals of our study was to investigate whether and how decisions in experimental situations are affected by the appearance of the interaction partner. In our experiment, each decision had to be taken three times, once in an anonymous setting and two more times in a “personalised” setting, after seeing a short silent video of the interaction partner. As shown in Supplementary Table S3, individuals were, to a certain extent, consistent in their decisions. For the non-binary decisions, we quantified consistency by the intraclass correlation coefficient: this ranged from 0.518 for the trustworthiness decision to 0.820 for the generosity decision. For the binary decisions, we quantified consistency by the percentage of cases where all three decisions were identical: this percentage ranged from 42.2% for the coordination decision to 82.8% for the punishment decision.

Despite this consistency, we found a clear effect of the interaction partners’ appearance on the behavioural outcomes. For each subject shown in a silent video in the personalisation setting, we asked a subset of the other participants to rate a picture showing the neutral face of the subject on a five-point scale (from very low to very high) regarding the following eight attributes: generous, trustworthy, rational, risk-taking, greedy, angry, happy, and attractive. The agreement among raters (as quantified by intraclass correlation coefficients; see “Methods”) ranged from 0.505 (for ‘greedy’) to 0.860 (for ‘attractive’) (see Supplementary Table S4), values that are generally considered high enough to use the average attribute scores for further analysis^35^.

**Figure 3:**
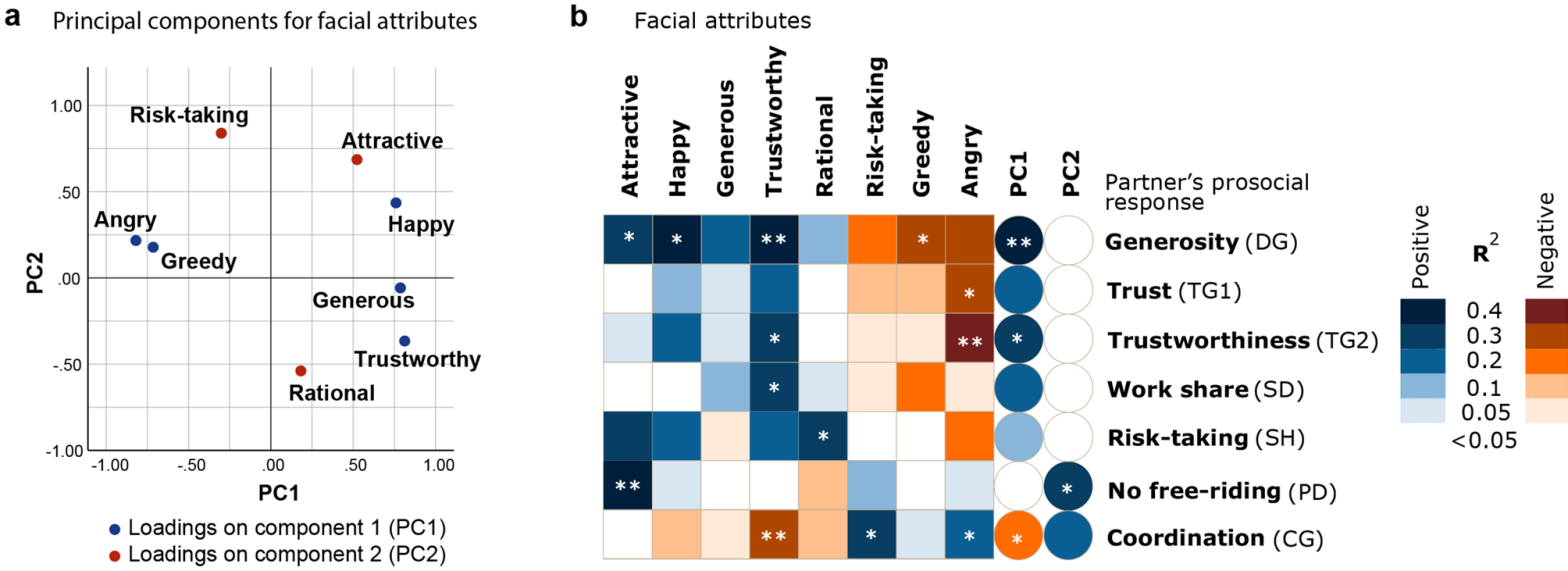
Effect of the interaction partner’s facial appearance on prosocial behaviour. (**a**) Principal Component Analysis of the eight facial attribute scores. PC1 (variance explained: 43.6%): happy, generous, trustworthy, not angry, not greedy; PC2 (variance explained: 23.4%): risk-taking, attractive, not rational. (**b**) Association between the subjects’ facial appearance (columns) and the behaviour elicited in their interaction partners in seven decision situations (rows). Facial appearance is quantified by the face judgement scores regarding eight attributes (attractive, happy, generous, trustworthy, rational, risk-taking, greedy, angry) and the scores for their principal components (PC1, PC2). The heat map gives a pictorial representation of the R^2^ values for positive and negative associations, based on linear regression models with a single predictor (see “Methods” for details on statistics, and Suppl. Table S5 for numerical values). Significant associations: *p < 0.05, **p < 0.01. The decision situations related to fairness (UG1), demand (UG2), and punishment (PG) are not shown, as the behaviour in these situations was not significantly associated with any of the facial scores (see Suppl. Table S5).

Figure 3a shows the results of a Principal Component Analysis of the eight facial attribute scores. Principal Component 1 (PC1) explains 43.6% of the variance and is characterised by positive loadings on the facial attributes ‘happy’, ‘trustworthy’, and ‘generous’, and negative loadings on ‘angry’ and ‘greedy’. Accordingly, PC1 seems to represent a prosocial (generous, trustworthy, non-greedy) and at the same time cheerful (happy, not angry) appearance. Principal Component 2 (PC2) explains 23.4% of the variance and combines positive loadings on ‘attractive’ and ‘risk-taking’ with a negative loading on ‘rational’. Thus, PC2 seems to represent a venturesome and attractive type of appearance.

To assess how the appearance of the interaction partners affects behaviour, we first investigated whether and how shifts in behaviour in the decision situations (i.e., changes in behaviour between personalised and anonymous setting) were related to the facial appearance of their interaction partner. The results, which are presented in Supplementary Figure S2, reveal that facial attributes related to prosocial (‘generous’, ‘trustworthy’, ‘not greedy’) and positively emotional (‘happy’, ‘not angry’) dimensions (summarized by PC1), as well as to attractiveness induced shifts towards generosity in the Dictator Game and towards trustworthiness in the Trust Game. Interestingly, the shift in behaviour tended to be in the opposite direction regarding work share in the Snowdrift Game, which could mean that in this context subjects tried to exploit prosocially looking partners by investing less in the common task. As explained in the legend of Figure S2, the behavioural shift analysis could, for statistical reasons, not be done for the five game-like situations with binary decisions.

To obtain a more complete picture, we therefore analysed the effect of facial appearance on behaviour in the personalised setting, without comparing this behaviour to that in the anonymous condition. Now, all ten game-like situations could be included in the analysis. Figure 3b shows that the facial appearance of the interaction partner has a systematic effect on prosocial behaviour in the majority of the game-like situations (7 out of 10). In general, individuals with a prosocial and cheerful appearance (i.e., with facial attributes that have a positive loading on PC1) had a positive effect on the prosocial behaviour of their interaction partners (a higher generosity, more trust and trustworthiness, a higher work share, and a higher tendency to take cooperation risks), while individuals with an appearance associated with angriness, greed, or risk-taking had the opposite effect. The effect of the interaction partner’s facial appearance is most pronounced in the case of generosity and weaker in the case of other prosocial tendencies (such as the willingness to take on a higher work share). Of all facial attributes, ‘trustworthiness’ has the most pronounced effect on the partner’s willingness to exhibit prosocial behaviour. Interestingly, the effect of the interaction partners’ facial appearance on prosocial tendencies was markedly different in the case of the Prisoner’s Dilemma game (PD). Here, a positive loading on PC2 (and, in particular, the facial attributes ‘attractive’ and ‘risk-taking’) was associated with prosocial behaviour (no free-riding) in the interaction partner. Cooperation in the PD is a risky choice; therefore, this result might indicate that participants only dare to cooperate if the interaction partner looks venturesome and attractive. The coordination game (CG) is another special case. Here, a prosocial and cheerful appearance (i.e., a positive loading on PC1) had a negative effect on choosing the ‘partner-preferred option’ (which we classified as ‘prosocial’), while an ‘angry’ or ‘risk-taking’ appearance had a positive effect. Upon closer inspection, this outcome makes perfect sense: if the interaction partner ‘indicates’ by their facial appearance that they are likely to take the (‘prosocial’) partner-preferred option, it is not ‘antisocial’ at all to choose the self-preferred option, because otherwise both partners would get a zero payoff. Similarly, it is neither pro- nor antisocial to go for the partner-preferred option if the interaction partner ‘indicates’ that they will decide on the self-preferred option.

Hence, our results suggest that facial traits are perceived similarly by different people and that a prosocial and cheerful appearance (a positive loading on PC1) induces a prosocial response in social situations related to generosity, trust, trustworthiness, and risky cooperation and an adequate response in the context of coordination. In contrast, a venturesome and attractive appearance (a positive loading on PC2) has a positive effect on the interaction partner’s tendency to cooperate in the Prisoner’s Dilemma game. We did not find any significant effect of personalisation in the other three decision situations, i.e., regarding fairness (UG1), demand (UG2), and punishment (PG).

### Are partners eliciting prosocial behaviour prosocial themselves?

**Figure 4:**
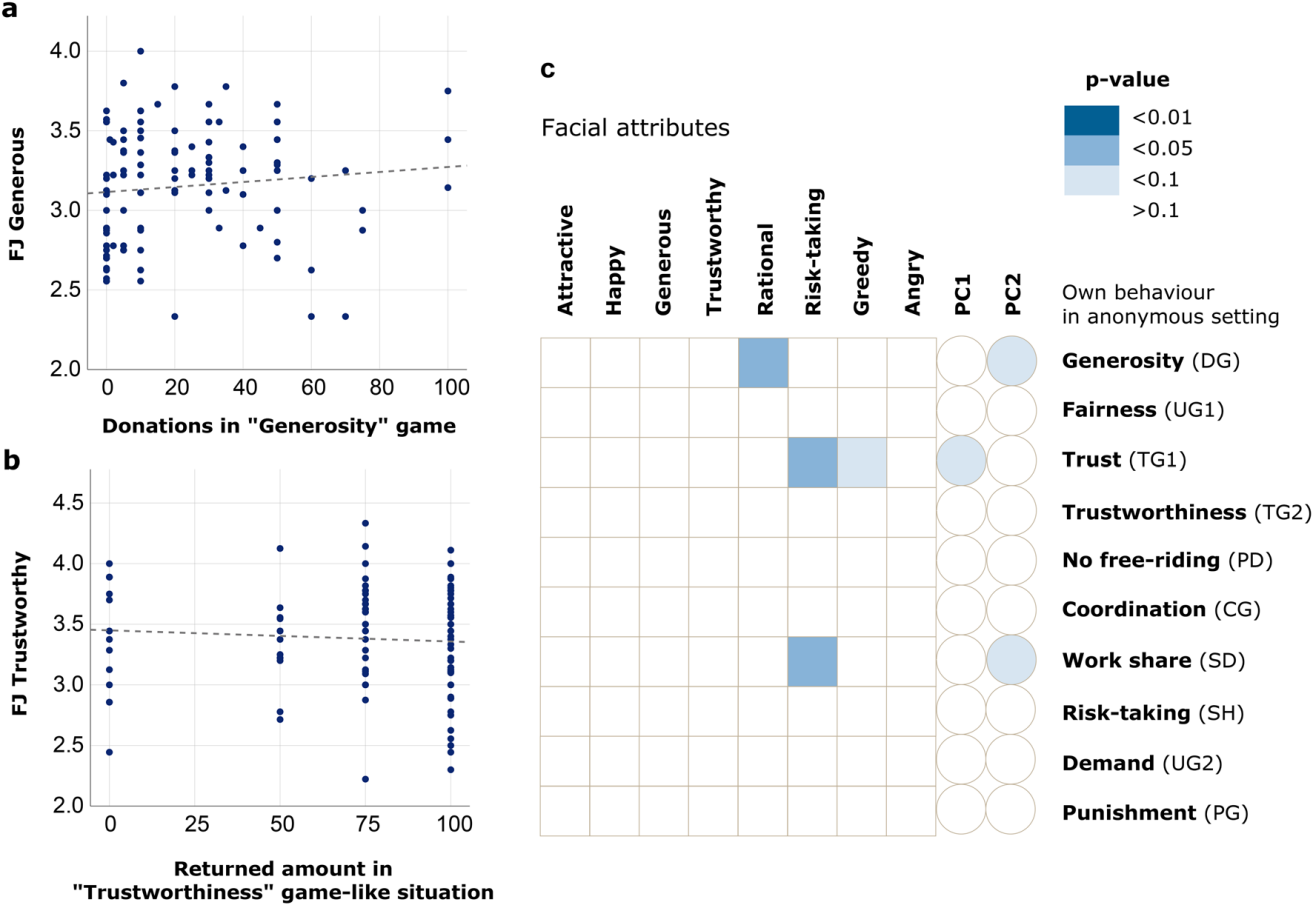
Relationship between facial attributes and prosocial behaviour. (**a**) Association between the scores for the facial attribute ‘generosity’ and the amount donated by the judged subjects in the anonymous version of the ‘Generosity game’ (DG). Linear regression analysis: N = 118, R^2^ = 0.011, B = 0.002, *p* = 0.254. (**b**) Association between the scores for the facial attribute ‘trustworthiness’ and the number returned points in the anonymous version of the ‘Trustworthiness game’ (TG2). Linear regression analysis: N = 118, R^2^ = 0.005, B = − 0.001, *p* = 0.434. (**c**) Heat map of the p-values of 100 tests addressing the statistical association between each of ten facial scores (columns: the eight facial attributes and the scores for PC1 and PC2) and behaviour in the anonymous version of the ten decision situations in our experiment (rows). The number of significant *p*-values (three for *p* < 0.05; four for *p* < 0.10) does not exceed the number of type II errors to be expected. For continuously scaled games (DG, SD, UG1, UG2, TG2), the scores of the subjects’ facial attributes and their PCs were regressed upon the subjects’ own decisions in the games (in points). For games with binary decisions (TG1, PD, SH, CG, PG) differences were estimated with a Student’s t-test.

Given the pronounced effect of the interaction partners’ facial appearance on the prosocial behaviour of our subjects, the question arises whether facial appearance is an indicator of pro- or antisocial behaviour. Does, for example, a prosocial and cheerful appearance (a positive loading on PC1) elicit prosocial behaviour because such an appearance is associated with prosocial tendencies? To address this question, we assessed whether there was a correspondence between the facial scores of the subjects classified for this and the actual behaviour of these subjects in our experiment. The results are shown in Fig. 4. Figure 4a demonstrates that there is no significant association (*p* > 0.25) between the degree of ‘generosity’ (the number of points donated to a stranger) in the Dictator Game and the face judgement regarding the attribute ‘generous’. Likewise, Fig. 4b shows that the degree of ‘trustworthiness’ (the number of points returned to the trustor) in the Trust Game is not associated with the face judgement regarding the attribute ‘trustworthy’ (*p* > 0.43). Figure 4c shows, more generally, that the associations between face judgements and the subjects’ actual behaviour were typically not significant. In fact, the number of significant test results did not exceed the number of type II errors to be expected when executing 100 tests. The associations reported in Fig. 4 were based on the anonymised version of the games. When repeating the analysis by also including the behaviour in the personalised version of our experiment, we obtained very similar results (see Supplementary Figure S3). We conclude that—although our subjects’ facial appearance had a clear effect on the decisions of their interaction partners—this appearance was not associated with the subject’s actual behaviour in the experimental games.

## Discussion

Thousands of experimental studies on human cooperation have been conducted in the four decades following the publication of Axelrod’s influential book on ‘The Evolution of Cooperation’^36^. In the initial period, the focus was on social dilemmas (like the Prisoner’s Dilemma Game or Public Goods Games), and it took quite a while before other aspects of cooperation than free-riding started to receive serious consideration. Still, there are few attempts to investigate a whole variety of cooperation aspects in a single study. Here, we report on such a study, where the same subjects had to make decisions in ten experimental situations that all focus on different aspects: generosity, trust, trustworthiness, fairness, demand, division of labour, coordination, propensity for free-riding, taking risks associated with cooperation, and prosocial punishment. In line with many earlier studies^12–15^, our experiment reveals considerable inter-individual variation in the behaviour in each of the cooperative contexts investigated.

Moreover, our results show that individuals tend to behave consistently in different kinds of prosocial situations, which is in line with other studies, revealing individual variation and consistency in human cooperative and free-riding predispositions^14–16,20^, social value orientation^12^, and social learning^19,23^. In addition, our study also demonstrates that different aspects of cooperativeness are correlated with each other. Animal behaviour scientists call a set of correlated behavioural tendencies a ‘behavioural syndrome’, and syndromes are considered an important part of ‘(animal) personalities’^37,38^. In animal behaviour, personality differences are determined on the basis of direct behavioural observations, while in psychology, personality structure is usually classified on the basis of self-assessments (introspection)^38^. Our approach (and that of similar studies like Refs.^12,13,23^) is more similar to that of animal behaviour research, where the actual behaviour in a whole suite of functionally related situations is used to unravel the principles underlying the ‘architecture of behaviour’.

In our study, subjects that exhibited trustworthiness in the Trust Game also tended to be generous, fair, trusting others, and abstain from free-riding. Prosocial punishment, demand, and risk-taking were not part of this ‘cooperativeness syndrome’. As shown in Fig. 2, all these results are remarkably similar to the findings of an earlier study by Peysakhovich and colleagues^13^, despite the fact that both studies differ in many respects, such as the experimental setting (online versus on-site), the exact nature of the games, the narratives presented to the participants, and the payoffs. In this regard, it is worth mentioning an even earlier study by Yamagishi et al.^21^ conducted in Japan. The results of that study (where the participants made anonymous decisions in the Prisoner’s Dilemma Game, the Trust Game, the Dictator Game, and the Ultimatum Game) also demonstrated that prosocial decisions across these games are positively correlated with each other, while the tendency to reject unfair offers in the Ultimatum Game (demand in our study) was not correlated to various behavioural tendencies in the other games (except for a weak negative correlation with cooperation in the Prisoner’s Dilemma). All in all, the agreement of our findings with those of earlier studies makes us confident that the observed patterns are quite robust, although it would of course be welcome to scrutinise them even more, e.g. in a more naturalistic setting or with non-WEIRD (Western, educated, industrialised, rich and democratic) participants^39^ with more diverse socio-economic and cultural backgrounds. Anyway, the first, ‘anonymous’ part of our study may be viewed as a contribution to countering the reproducibility crisis in the social and behavioural sciences^40,41^.

Although the behaviour in anonymous settings provides interesting insights, the main objective of our study was to investigate how personalisation affects prosocial behaviour. We wanted to know whether the presentation of a short silent video of the interaction partner is sufficient to affect cooperative behaviour in a consistent way. Our study shows that such a mild form of personalisation has indeed a systematic and repeatable effect on cooperative decisions. Participants whose neutral facial photographs received high scores (by a separate assessment panel) for attributes like ‘generous’, ‘trustworthy’, ‘happy’, and ‘attractive’, had a positive effect on prosocial behaviour in their interaction partners, while those with high scores for ‘angry’, ‘greedy’, and ‘risk-taking’ had a negative effect. Quite a number of studies investigated the effect of attractiveness on decisions in the Prisoner’s Dilemma Game, the Public Goods Game^42–45^, and the Trust Game^45,46^. These studies found that people tend to trust and cooperate with attractive-looking individuals, although such individuals do not actually meet the expectations of their trustors^44–46^, but see Ref^47^. Our results confirmed that attractiveness elicits a positive response in the context of cooperation in the Prisoner’s Dilemma (as well as an increase in generosity in the Dictator Game), but other facial attributes (like perceived trustworthiness) of the interaction partner had a stronger and less specific effect than attractiveness. Therefore, we do not think that our findings are just side effects of attractiveness.

It is plausible to assume that personalisation affects prosocial behaviour because even a neutral picture may reveal subtle information on the intentions and/or the prosocial tendencies of one’s interaction partner. Various studies do indeed report evidence that facial features allow the prediction of human prosocial behaviour^31,48–50^. For example, artificial neural networks can be trained to predict (to a certain extent) Big Five personality traits on the basis of individual portraits^50^. However, various other studies arrived at the opposite conclusion. For example, a recent study^51^ concludes that people cannot predict human trustworthiness on the basis of static facial appearance when this is the only available information. Another study reports that trustworthy-looking individuals receive a positive response regardless of their ‘good’ or ‘bad’ history^52^. Our results are in line with those latter studies. We did not find evidence that face judgements like ‘generous-looking’ or ‘trustworthy-looking’ are associated with the actual behaviour (like acting generously in a donation context or acting trustworthily in a trust context) of the judged individuals. Moreover, happy-looking or attractive-looking individuals elicited more prosocial behaviour in their interaction partners (Fig. 3), but a happy or attractive look was not associated with a higher degree of prosociality in any of the ten decision situations tested (Fig. 4). In other words, in our experiment, the higher prosociality elicited by the facial features of some of the participants was not justified by a higher prosocial attitude of these participants.

Although we did not find evidence for the ability to predict prosocial attitudes on the basis of facial features, such prediction might be possible in real-life situations. It is worth noting that all studies reporting a lack of predictability of cooperativeness on the basis of visual cues, including our own, used young people (with a similar background) as participants. The results of such studies may not be representative for other age classes. The increase in general cooperativeness and prosociality with age is a well-established phenomenon in humans^22,53^. This may be due to age-related differences in goals and priorities, such as a greater focus on intrasexual competition and short-term mating behaviour in younger people, which may compromise prosocial qualities^54,55^. Such age-related priorities may cause biases in the perception of visual cues, which, in turn, may distort the correct identification of the interaction partners’ prosocial tendencies. This issue should be studied in more detail in the future, by conducting experiments with subjects of a wider age range.

We are aware that our study has other limitations. For example, we pooled the results of male and female participants in order to increase the sample size and the power of statistical tests. Such pooling is common practice if, as in our study (for the case of anonymous interactions), no significant sex differences in the distributions of decisions are observed. In a separate article^56^, we show that, nevertheless, there are interesting sex differences regarding trust and trustworthiness. Another limitation is the fact that the subjects of our study had to make three decisions in each experimental situation. In each case, the anonymous decision was taken before the two personalised decisions. This order was chosen to prevent that seeing an interaction partner’s video would influence a subject’s decision in the anonymous context (which served as our baseline). But it implies that our study might suffer from order effects, as reported in the repeated Prisoner’s Dilemma Game and the repeated Public Goods Game, where the tendency to cooperate decreases with time^57,58^. However, we did not find evidence for such order effects, with the exception of a minor and inconsistent decrease in cooperation in the Prisoner’s Dilemma Game and the Snowdrift Game. Adding the order of interaction as a cofactor in the regression analyses of Fig. 3 did not change any of our conclusions.

In the personalisation part of our experiment, the interaction partners were presented to the subjects through short silent videos. In contrast, the face judgements were, for logistic reasons, made on the basis of neutral facial images. Possibly, the judgements of participants on the basis of videos instead of pictures would have resulted in stronger effects, but it is reassuring that, even with this limitation, the outcome of our study was rather clear-cut: facial characteristics of the interaction partners had a systematic effect on prosocial behaviour of our participants. In our experiment, such a response did not increase cooperation benefits, as facial appearance was not indicative of actual prosocial behaviour.

To sum up, the results of our study (a) confirm and advance earlier findings that prosocial behavioural tendencies in a wide variety of contexts constitute a ‘cooperativeness syndrome’, but the tendency to punish non-cooperative individuals is not part of this syndrome; (b) reveal a multi-faceted effect of partners’ facial appearance on behaviour in different situations related to cooperation and coordination; (c) contribute to the emerging evidence that (at least among relatively young individuals from WEIRD societies), the facial appearance of an individual is not a reliable predictor of the individual’s prosocial behaviour, regardless of the facet of prosociality studied.

## Methods

### Participants

Initially, we aimed at 100 male and 100 female participants but could recruit only 181, of which 176 participated in all experimental procedures (87 females, 89 males). All participants were 18–30 years old, with male subjects being slightly older (median = 22 y) than females (median = 21 y). The majority of participants were students of the University of Groningen (mostly from the Faculty of Science and Engineering and the Faculty of Behavioural and Social Sciences) and of Dutch, Belgian, and German origin. The study received approval from the Ethical Committee Psychology (ECP) of the University of Groningen (Research Code: 16250-O). Prior to the experiment, all subjects signed informed consent.

### Experimental procedure

Participants were asked to show up on two different days. On the first day, a silent video of each participant was recorded (20 s. neutral talk to the camera), and face photographs were taken (full-face, looking at the camera with neutral facial expression). Videos and photographs were taken under standard conditions. On the second day, the interaction part of the experiment and the rating of the photographs were conducted in large computer rooms at the University of Groningen. Subjects were invited in groups of 15–21 same-sex participants; they were not allowed to communicate with each other and were asked to address all questions only to the experimenter. Each participant was seated at a personal computer, separated from neighbours by vertical desk dividers. They were informed that all decisions would be treated anonymously and nobody else would know their decisions at any step of the experiment.

All experiments in the interaction were implemented in the Survey Monkey Audience online form, which included both the option of face judging and the experimental games. All subjects had to make decisions in eight games. As the roles of the players in two of these games are asymmetric, each subject had to make choices in ten decision situations (see Tables 1 and 2). In each of these situations, the same decision had to be taken three times: first without any information on the interaction partner (anonymous condition), and second and third after having seen a silent 20 s. video of their interaction partner (personalised condition). To prevent transfer effects, the games were separated by letting the participants fill out questionnaires with unrelated questions. Each participant saw 14 unique videos during the experiment: two interaction partners in (i) the Dictator Game; (ii) the Prisoner’s Dilemma game; (iii) the Snowdrift Game; (iv) the Coordination Game; (v) the Trust Game (the same interaction partner for the ‘trust’ and the ‘trustworthiness’ decision); (vi) the Ultimatum Game (the same interaction partner for the ‘fairness’ and the ‘demand’ decision); and (vii) the Stag Hunt Game and the Punishment Game (the same interaction partner was shown in the Punishment Game, as punishment was related to the decisions in the Stag Hunt Game). In total, 140 randomly chosen videos (70 male and 70 female) were used in the personalised setting. Each video was planned to be shown 20 times in one of the seven ‘personalisation’ situations sketched above, but as participation was somewhat lower than initially expected, each video was actually shown to 17 ± 2 different participants. In each personalised interaction, the participant was asked whether they knew the partner shown in the video in person. When the answer was affirmative, the experiment continued, but the case was excluded from further analysis. In the Coordination Game and the Trust Game (see Ref ^56^ for a detailed description and analysis), the above procedure was slightly extended: in addition to the two personalised same-sex interactions, each participant also had two personalised opposite-sex interactions. Here, we only report on the same-sex interactions.

In total, 2464 (= 176 · 14) videos were displayed during the experiment. 2446 of the corresponding interactions entered the analysis, as 18 interactions were excluded due to the personal acquaintance of the interaction partners. Two participants whose videos and facial photographs had been taken on the first day of the experiment did not show up on the second day. Due to logistic reasons, their videos were still shown to other participants on the second day of the experiment (one in the Dictator Game and one in the Coordination Game), and their facial photographs were rated. However, these two cases dropped out from the corresponding analyses in Figs. 1, 2 and 4.

To prevent learning effects, the participants did not get immediate feedback on the outcome of any of their interactions; they only received a cumulative payoff at the end of the whole experiment. This had the logistic advantage that the games did not have to be real-time interactive. First, the decisions of all participants in the various decision situations were recorded. At the end of the experiment, payoffs were assigned to these decisions, by matching each decision of a participant with a decision of the participant’s interaction partner. In the anonymous setting, the interaction partner was chosen randomly (from the same sex), while in the personalised setting, the partner was the person shown on the video. Under personalised condition, the interaction partner’s decision was the decision that the partner had made in the anonymous setting of the same game, as we assumed that these decisions best reflect the basal behavioural predispositions of the partner. Subsequently, the payoffs were calculated according to the game rules (see below) and summed up with the payoffs from all other experimental games. From the start of the experiment, participants were informed that the outcomes of interactions (and the resulting payoff calculations) would be based on the decisions their interaction partners made in the anonymous setting.

The participants did not receive any information about the exchange rate between the currency used in the experimental games (points) and their final payoff in Euros; they were only informed about their overall payoffs (in Euros) at the end of the experiment. Participants did not receive any feedback on the payoffs (neither in points nor in Euros) of the separate interactions. At the stage of recruitment, the subjects were told that the average *per capita* payoff for the whole experiment would be around 40 €, but that the actual payoff would strongly depend on individual performance. Therefore, participants were highly motivated to optimise their decisions.

A detailed description of the experimental procedures can be found in our extensive study protocol^34^.

### Face judgements

Of the neutral facial pictures taken of the participants, 120 (60 females, 60 males) were randomly selected to be presented for face judgements. These were randomly distributed over ten sets of six female pictures and ten sets of six male pictures. During the experiment, each participant was presented with one set of six same-sex pictures and asked to score them on a five-point scale (from very low to very high) regarding eight attributes: generous, trustworthy, rational, risk-taking, greedy, angry, happy, and attractive. These attributes were chosen to cover both prosociality-related traits (generous, trustworthy, rational, risk-taking, greedy) and more general traits, which might also influence prosocial responses. Each participant was asked whether they knew the individual shown in a photograph in person; if so, the case was excluded from the analysis (four male and three female cases). Given the initially expected participation of 100 male and 100 female subjects, we assumed that each picture would be rated by ten same-sex participants. However, due to lower participation (N = 176), each male set of six pictures was on average judged by 8.9 participants (from 7 to 10), and each female set of six pictures was on average judged by 8.7 participants (from 7 to 11). Participants who rated the photographs were never matched as interaction partners with the subjects shown in the photographs.

### Experimental games

The participants of our experiment faced ten cooperation-related decision situations that were based on eight classical economic games. Table 2 summarises the rules and the payoff structure of each game. To achieve a compromise between internal and external validity of our experimental results, all decision situations were embedded in a real-life story. Table 1 in the main text provides short descriptions of these narratives. The full narratives provided to the participants can be found in the study protocol^34^.

### Statistical analysis

All statistical analyses were conducted in SPSS v. 26.

For the analysis of decisions across different experimental situations, we used categorical Principal Component Analysis (catPCA) followed by varimax rotation as a standard build-in procedure for dimension reduction in SPSS. The catPCA allows for handling variables of different measurement level (binary, categorical, and numeric) within the same analysis. In this method, while performing dimension reduction, categories of variables with nominal and ordinal levels are transformed into numeric values (excluding missing values and obvious outliers), using the so-called optimal scaling procedure. Technical details and references can be found in the corresponding literature^59^. The correlation matrix of the transformed variables was used to estimate the consistency of individual decisions across different games.

For testing between-rater agreement in judging facial photographs on each of the attributes, we used Intraclass Correlation Coefficients analysis (ICC). This analysis allows to quantify the agreement between raters judging the same face. The number of subjects was equal to 120 (which is the number of judged portraits), and the number of ratings per portrait varied from 7 to 11. Since our design involved different sets of raters judging different subsets of facial photographs, the ICC analysis was based on meta rating, absolute agreement, and a one-way random-effects model (ICC, 1, k), where the number k of raters varied from 7 to 11^60^. As the ICC values for the average measurements were sufficiently high (Supplementary Table S4), we used the mean scores of the facial attributes in all further analyses.

To understand how seeing a silent video of the interaction partner could influence the prosocial behaviour of participants, we conducted two kinds of analysis. First, we analysed how the behaviour in the personalised setting differed from that in the anonymous setting and how the shift in behaviour was related to the facial characteristics of the interaction partner in the personalised setting. For statistical reasons, this analysis could not be done for the five game-like situations with binary choices. Therefore, the results are somewhat limited and presented in Supplementary Figure S2. The corresponding methodology is explained in the legend of that figure. Second, we analysed how the behaviour in the personalised setting was related to the facial appearance of the interaction partners. Twenty videos were used for a given game-like situation. Each video was displayed to 17 (± 2) different participants. The facial appearance of persons in these videos was quantified by the above-mentioned judgement of their facial pictures by a separate panel. For each of the 8 facial attributes, this yielded an average score ranging between 1 and 5. The behavioural response elicited by each of the 20 persons in the given game-like situation was quantified by the average decision made by the 17 (± 2) subjects who interacted with this person in the personalised setting. In the case of the five situations with binary decisions, the two options were encoded as ‘0’ or ‘1’, yielding an average ‘behavioural response’ value ranging between 0 and 1. Finally, we analysed for each of the game-like situations how the average behavioural response value of each of the 20 persons on the videos was related to the 8 facial attribute scores of that person. This was done by using linear regression models with a single predictor variable (the score regarding the facial attribute under consideration) and a single response variable (the ‘behavioural response’ score for the game situation under consideration).

### Supplementary Information

Supplementary Information.

## Data Availability

The dataset supporting the present study can be accessed through public repository: 10.34894/VAEJSF
