# Supplementary material for: Unravelling the many facets of human cooperation in an experimental study
Source: Sci Rep. 2023 Nov 10;13:19573. doi: 10.1038/s41598-023-46944-w (PMC10638426; doi:10.1038/s41598-023-46944-w)
Supplement: Supplementary file 1 — Supplementary Information. [file 41598_2023_46944_MOESM1_ESM.pdf]

Unravelling the many facets of human cooperation in an experimental study

V.V. Rostovtseva, M. Puurtinen, E. Méndez Salinas, R.F.A. Cox, A.G.G. Groothuis, M.L. Butovskaya, F.J. Weissing

Supplementary Figures

Supplementary Figure S1. Associations between decisions in different experimental situations: Effect of (a) exclusion or (b) inclusion of the Fairness decision of the Ultimatum game on the catPCA analysis in Figure 2b.

Supplementary Figure S2. Effect of the subjects' facial appearance on the shift in behaviour of their interaction partners.

Supplementary Figure S3. Relationship between facial attributes and prosocial behaviour: Effect of pooling anonymous and personalised decisions on the test results in Figure 4c.

Supplementary Tables

Supplementary Table S1. Correlation matrix underlying the heatmap in Figure 2a: Associations between decisions in different experimental situations in the anonymous setting.

Supplementary Table S2. Economic games used in the study of Peysakhovich et al. (2014; ref 13).

Supplementary Table S3. Within-game consistency of decisions across anonymous and personalised conditions.

Supplementary Table S4. Between-rater agreement in judging faces for each attribute.

Supplementary Table S5. Statistical analyses underlying the heatmap in Fig. 3b: Effect of the interaction partner's facial attributes on the behaviour in the ten experimental decision situations.

Supplementary Figure S1. Associations between decisions in different experimental situations: Effect of (a) exclusion or (b) inclusion of the Fairness decision of the Ultimatum game on the catPCA analysis in Figure 2b.

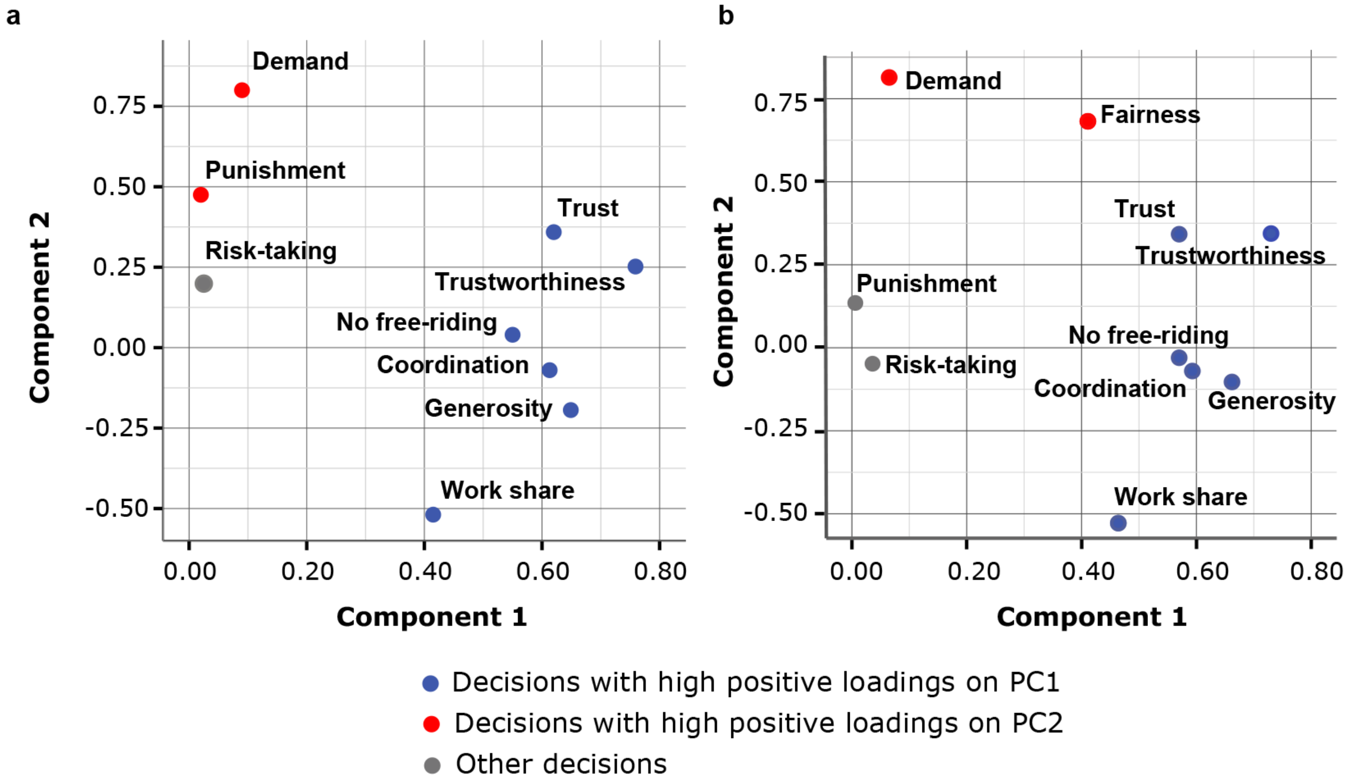

Principal component loadings of the decisions from the catPCA of our experiment (N = 168). The panels show two variants of Fig. 2b of the main text. **a.** Results of the catPCA when the Fairness decisions in the Ultimatum Game are excluded, as in Fig. 2b (to facilitate the comparison of our results with those in reference 13). Variance explained: Comp 1 = 25.2%, Comp 2 = 15.4%; Total = 40.6%. **b.** Results of the catPCA when the Fairness decisions are included in the analysis. Variance explained: Comp 1 = 23.6%, Comp 2 = 18.8%; Total = 40.4%.

Addition of the Fairness part does not markedly change the composition of Comp 1 composition, but the composition of Comp 2 is changed substantially. This illustrates the well-known fact that Principal Component Analyses are sensitive to the number of data dimensions.

Labels refer to the following decision situations: generosity: Dictator Game; trust: decision 1 of Trust Game; trustworthiness: decision 2 of Trust Game; no free-riding: Prisoner's Dilemma Game; coordination: Coordination Game; workshare: Snowdrift Game; risk-taking: Stag Hunt Game; fairness: decision 1 of Ultimatum Game; demand: decision 2 of Ultimatum Game; punishment: Punishment Game.

Supplementary Figure S2. Effect of the subjects' facial appearance on the shift in behaviour of their interaction partners.

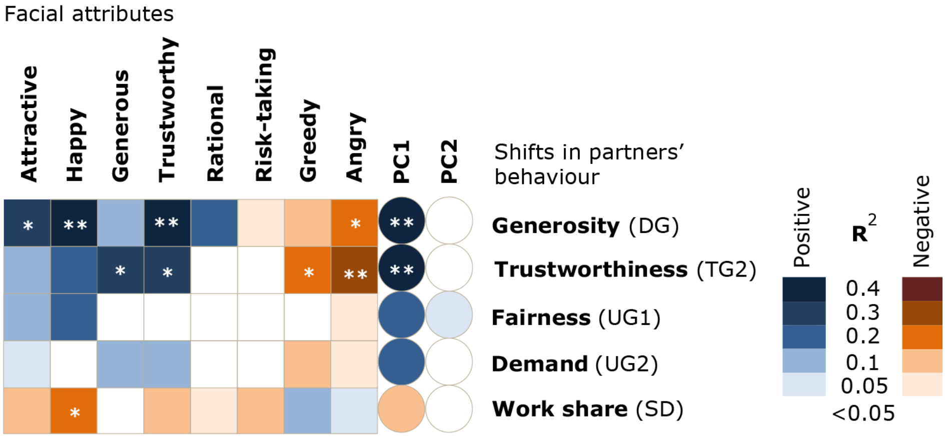

Association between the subjects' facial appearance (columns) and the behavioural shifts elicited in their interaction partners in five decision situations (rows). The heat map gives a pictorial representation of the R^2 values for positive and negative associations, based on linear regression models with a single predictor. Significant associations: *p<0.05, **p<0.01.

Facial appearance is quantified by the face judgement scores regarding eight attributes (attractive, happy, generous, trustworthy, rational, risk-taking, greedy, angry) and the scores for their principal components (PC1, PC2). For each subject, the shift in their partners' behaviour was calculated as follows. Each partner received a score of +1, when the partner's decision in the interaction with the given subject changed in the positive direction when compared to the anonymous setting, a score of -1 if it changed in the negative direction, and a score of 0 if it did not change. These scores were subsequently averaged over all partners who interacted with the given subject in a personalised setting. Finally, the mean scores were regressed upon the subjects' face judgement scores per attribute.

The five binary decision situations had to be left out of the analysis, as in these games the scores for a shift in behaviour are intrinsically biased: if the decision was a '0' in the anonymous setting, it could only change in the upward direction in the personalised setting; if it was a '1', it could only change in the downward direction.

Supplementary Figure S3. Relationship between facial attributes and prosocial behaviour: Effect of pooling anonymous and personalised decisions on the test results in Figure 4c.

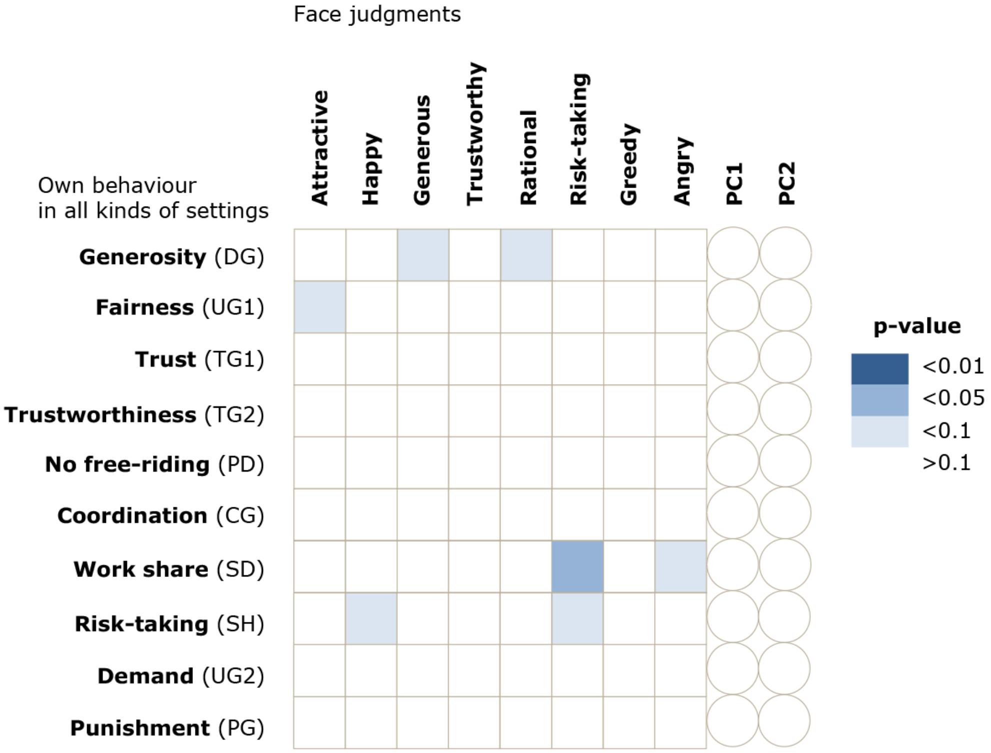

From Fig. 4 in the main text, we concluded that the results of our experiment do not provide evidence for the hypothesis that facial attributes of an individual are associated with the individual's propensity for prosocial behaviours. The analysis in Fig. 4c was solely based on the decisions made under anonymous conditions. As the decisions under anonymous conditions were, to a certain extent, associated with the decisions under personalised conditions (see Supplementary Table S3), the power of the tests underlying Fig. 4c might be enhanced by pooling the three decisions made by each individual (one under anonymous and two under personalised conditions). The heat map above illustrates that pooling the three decisions in a given cooperation context does not produce clearer associations between facial attributes and prosocial decisions than the analysis in Fig. 4c.

Heat map of the p-values of 100 tests addressing the statistical association between each of ten facial scores (columns: the eight facial attributes and the scores for PC1 and PC2) and the pooled decisions (anonymous and personalised) in the ten experimental situations (rows). As in Fig. 4c, we conclude that the number of significant p-values (one for $p<0.05$; six for $p<0.10$) does not exceed the expected number of type II errors.

Generalized Estimating Equations accounting for repeated measurements were used: linear models, where scores for facial judgements were set as response variables, and decisions in the experimental situations were set as independent factors (for binary scaled decisions) or covariates (for continuously scaled decisions).

Supplementary Table S1. Correlation matrix underlying the heatmap in Figure 2a: Associations between decisions in different experimental situations in the anonymous setting.

	TG 1	TG 2	UG1	UG2	SH	PG	PD	SD	CG
**DG**	**0.218** *0.005*	**0.297** *<0.001*	**0.236** *0.002*	0.022	0.031	–0.016	**0.239** *0.002*	**0.252** *0.001*	**0.275** *<0.001*
**TG 1**		**0.526** *<0.001*	**0.330** *<0.001*	**0.203** *0.008*	0.034	0.038	0.137	0.081	**0.232** *0.003*
**TG 2**			**0.297** *<0.001*	**0.192** *0.012*	–0.042	0.015	**0.341** *<0.001*	0.100	**0.341** *<0.001*
**UG1**				**0.493** *<0.001*	–0.035	–0.048	**0.241** *0.002*	0.014	0.134
**UG 2**					–0.009	**0.182** *0.018*	0.029	**–0.192** *0.013*	0.044
**SH**						0.112	**0.205** *0.008*	–0.030	–0.102
**PG**							0.049	0.039	0.010
**PD**								0.147	**0.195** *0.011*
**SD**									0.145

Correlation matrix for the decisions in the experimental situations: Dictator Game (DG), Ultimatum Game (UG1, UG2), Trust Game (TG1, TG2), Prisoner's Dilemma (PD), Coordination Game (CG), Snowdrift Game (SD), Stag Hunt Game (SH), Punishment Game (PG). The top row of each cell shows the Pearson correlation coefficients (transformed through catPCA, N=168) of the corresponding pair of decisions. Any correlation coefficient with absolute value above 0.15 is statistically significant at the 0.05 level. Significant associations are **bold**, and the corresponding p-values are displayed in *italics* below the corresponding correlation coefficient.

Supplementary Table S2. Economic games used in the study of Peysakhovich et al. (2014; ref 13).

Aspect	Game	Game rules
Generosity	Dictator Game (DG)	One-sided decision to donate $0 \leq x \leq 50$ points to the interaction partner, keeping 100-*x* points for themselves.
Trust	Trust Game (TG1)	The trustor decides whether to entrust 50 points to the trustee. If entrusted, the number of points is tripled, and the trustee decides how many points (up to 150) to return to the trustor. Unreturned points are kept by the trustee.
Trustworthiness	Trust Game (TG2)	
Fairness	Ultimatum Game (UG1)	Player 1 makes a proposal on how to allocate 100 points between self and Player 2. [This part of the game was not analysed in ref. 13.]
Demand (MAO)	Ultimatum Game (UG2)	Player 2 indicates their "minimal acceptable offer (MAO)". Any offer of Player 1 below the MAO will be rejected (and both players receive nothing).
Free-riding	Public Goods Game (PGG)	Four participants start with a 100-point endowment each. Each player decides how many points (from 0 to 100) to contribute to a common project. The individual contributions are lost for the players. However, the sum of all four contributions is doubled and distributed evenly among the four players.
Risk-taking (competitiveness)	All-Pay Auction (AP)	Two players start with a 100-point endowment each. Each player decides how many points (from 0 to 100) to invest in the competition for a 100-point prize. The money invested is lost, but the player who invests more receives the 100 points (in case of a draw, the winner is assigned randomly).
Punishment	Second Party Punishment (2PP)	Two players start with a 100-point endowment each. In a first stage, each player decides whether to give up 30 points to increase the other player's endowment by 60 points (C) or not (D). In a second stage, each player can pay $0 \leq y \leq 14$ points to reduce the other player's payoff by 5•*y* points. The values of *y* can be made dependent on whether the other player chose C or D in stage 1.
Third-party punishment	Third-Party Punishment (3PP)	Two players are each endowed with 100 points. Player A can decide whether to 'take' from Player B. If A takes, then B loses 50 points while A gains 30 points. A third player, C, receives 20 points and can pay $0 \leq y \leq 20$ points to reduce A's payoff by 5•*y* points if A decided to 'take' from B.

For comparative purposes, we use only the results of Experiment 1 from ref 13. This part involved 576 participants recruited using Amazon's Mechanical Turk, with no sex or nationality of the participants specified. Participants earned 50 cents for accepting the online survey. They then made decisions in the above-listed economic games. After completing the study, one role in one of these games was chosen at random, participants were matched accordingly and then paid based on their earnings in that game (up to $2, depending on which game was picked and what the players' decisions were).

Supplementary Table S3. Within-game consistency of decisions across anonymous and personalised conditions.

Type of decision	Game	Trait	ICC
Continuous	Dictator Game (DG)	Generosity	0.820
	Trust Game 2 (TG 2)	Trustworthiness	0.518
	Snowdrift Game (SD)	Work Share	0.642
	Ultimatum Game 1 (UG 1)	Fairness	0.777
	Ultimatum Game 2 (UG 2)	Demand	0.872
			**% absolute agreement**
Binary	Trust Game 1 (TG1)	Trust	70.9
	Prisoner's Dilemma (PD)	Free-riding	56.7
	Stag Hunt Game (SH)	Risk-taking	73.7
	Punishment Game (PG)	Punishment	82.8
	Coordination Game (CG)	Coordination	42.2

The table displays the degree of within-individual agreement between the three decisions (one in the anonymous and two in the personalised settings) in each experimental situation. For continuously-scaled games, consistency is measured in terms of intraclass correlation coefficients (ICC); for binary-scaled games, consistency is quantified by the percentage of absolute agreement, i.e., the percentage of cases where all three decisions were the same.

Supplementary Table S4. Between-rater agreement in judging faces for each attribute.

Attribute	ICC	95% Confidence Interval		Reliability
		Lower Bound	Upper Bound	
**Attractive**	0.860	0.820	0.890	excellent
**Risk-taking**	0.730	0.650	0.800	good
**Happy**	0.680	0.590	0.760	good
**Trustworthy**	0.670	0.426	0.669	good
**Angry**	0.660	0.560	0.740	good
**Generous**	0.550	0.425	0.660	fair
**Rational**	0.538	0.406	0.650	fair
**Greedy**	0.505	0.363	0.630	fair

For testing between-rater agreement in judging facial photographs on each of the attributes we used Intraclass Correlation Coefficients analysis (ICC). This analysis allows to quantify the agreement between raters judging the same face. The number of subjects was equal to 120 (which is the number of judged portraits), and the number of ratings per portrait varied from 7 to 11. Since our design involved different sets of raters judging different subsets of facial photographs, the ICC analysis was based on meta rating, absolute agreement, and a one-way random-effects model (ICC, 1, k), where the number k of raters varied from 7 to 11 (for details see ref. 54). As the ICC values for the average measurements were sufficiently high (Supplementary Table S4), we used the mean scores of the facial attributes in all further analyses. The measure of reliability was adopted from Cicchetti (1994).

Supplementary Table S5. Statistical analyses underlying the heatmap in Fig. 3b: Effect of the interaction partner's facial attributes on the behaviour in the ten experimental decision situations.

Game	Model	Facial attribute	B	R^2	p
DG[a]	1	**Attractive**	**7.914**	**0.332**	**0.015**
	2	**Happy**	**8.352**	**0.354**	**0.012**
	3	Generous	6.679	0.170	0.100
	4	**Trustworthy**	**7.693**	**0.381**	**0.008**
	5	Rational	6.284	0.132	0.152
	6	Risk-taking	-4.046	0.160	0.111
	7	**Greedy**	**-6.036**	**0.255**	**0.039**
	8	Angry	-4.282	0.152	0.122
	9	**PC1 (FJs)**	**3.360**	**0.429**	**0.004**
	10	PC2 (FJs)	-0.899	0.019	0.600
TG1[b]	1	Attractive	0.024	0.014	0.653
	2	Happy	0.083	0.083	0.261
	3	Generous	0.056	0.027	0.527
	4	Trustworthy	0.134	0.204	0.069
	5	Rational	0.034	0.015	0.636
	6	Risk-taking	-0.067	0.076	0.283
	7	Greedy	-0.113	0.081	0.267
	8	**Angry**	**-0.163**	**0.264**	**0.035**
	9	PC1 (FJs)	0.054	0.177	0.093
	10	PC2 (FJs)	-0.012	0.013	0.659
TG2[a]	1	Attractive	3.317	0.071	0.300
	2	Happy	7.097	0.168	0.102
	3	Generous	5.348	0.068	0.311
	4	**Trustworthy**	**9.208**	**0.263**	**0.035**
	5	Rational	1.905	0.013	0.661
	6	Risk-taking	-2.805	0.037	0.462
	7	Greedy	-6.119	0.065	0.323
	8	**Angry**	**-11.685**	**0.373**	**0.009**
	9	**PC1 (FJs)**	**4.165**	**0.285**	**0.027**
	10	PC2 (FJs)	-0.082	<0.001	0.960
SD[a]	1	Attractive	0.869	0.021	0.565
	2	Happy	1.843	0.026	0.527
	3	Generous	3.482	0.099	0.204
	4	**Trustworthy**	**6.532**	**0.263**	**0.029**
	5	Rational	1.046	0.008	0.720
	6	Risk-taking	-2.257	0.067	0.300
	7	Greedy	-4.318	0.194	0.068
	8	Angry	-2.329	0.045	0.400
	9	PC1 (FJs)	2.140	0.198	0.065
	10	PC2 (FJs)	-0.479	0.014	0.641
SH[b]	1	Attractive	0.091	0.280	0.094
	2	Happy	0.108	0.183	0.189
	3	Generous	-0.049	0.038	0.566
	4	Trustworthy	0.085	0.179	0.195
	5	**Rational**	**0.205**	**0.437**	**0.027**
	6	Risk-taking	-0.006	0.001	0.921
	7	Greedy	0.028	0.011	0.763
	8	Angry	-0.093	0.159	0.236

	9	PC1 (FJs)	0.034	0.117	0.302
	10	PC2 (FJs)	0.005	0.003	0.879
PD[b]	1	**Attractive**	**0.180**	**0.365**	**0.008**
	2	Happy	0.071	0.027	0.512
	3	Generous	-0.070	0.021	0.570
	4	Trustworthy	0.051	0.017	0.610
	5	Rational	-0.174	0.148	0.115
	6	Risk-taking	0.110	0.126	0.148
	7	Greedy	-0.015	0.001	0.881
	8	Angry	0.092	0.067	0.299
	9	PC1 (FJs)	0.011	0.004	0.797
	10	**PC2 (FJs)**	**0.096**	**0.311**	**0.016**
CG[b]	1	Attractive	-0.002	<0.001	0.972
	2	Happy	-0.144	0.143	0.110
	3	Generous	-0.112	0.073	0.263
	4	**Trustworthy**	**-0.181**	**0.327**	**0.010**
	5	Rational	-0.117	0.103	0.180
	6	**Risk-taking**	**0.115**	**0.273**	**0.022**
	7	Greedy	0.106	0.056	0.331
	8	**Angry**	**0.133**	**0.281**	**0.044**
	9	**PC1 (FJs)**	**-0.065**	**0.218**	**0.044**
	10	PC2 (FJs)	0.053	0.156	0.095
UG1[a]	1	Attractive	0.312	0.009	0.707
	2	Happy	1.036	0.041	0.404
	3	Generous	1.307	0.068	0.279
	4	Trustworthy	0.892	0.038	0.426
	5	Rational	0.122	<0.001	0.943
	6	Risk-taking	-0.726	0.025	0.519
	7	Greedy	-1.159	0.056	0.330
	8	Angry	-0.125	0.001	0.888
	9	PC1 (FJs)	0.385	0.047	0.371
	10	PC2 (FJs)	-0.065	0.001	0.927
UG2[a]	1	Attractive	-0.377	0.003	0.837
	2	Happy	-0.041	<0.001	0.988
	3	Generous	0.672	0.004	0.804
	4	Trustworthy	-0.043	<0.001	0.986
	5	Rational	0.599	0.002	0.873
	6	Risk-taking	-0.634	0.004	0.799
	7	Greedy	-1.086	0.010	0.682
	8	Angry	0.558	0.005	0.776
	9	PC1 (FJs)	0.033	<0.001	0.973
	10	PC2 (FJs)	-0.475	0.006	0.761
PG[b]	1	Attractive	0.013	0.005	0.838
	2	Happy	0.015	0.003	0.875
	3	Generous	-0.067	0.060	0.469
	4	Trustworthy	0.075	0.116	0.305
	5	Rational	0.085	0.063	0.456
	6	Risk-taking	-0.044	0.049	0.515
	7	Greedy	0.029	0.009	0.776
	8	Angry	0.016	0.004	0.861
	9	PC1 (FJs)	0.004	0.001	0.915
	10	PC2 (FJs)	-0.020	0.032	0.598

Statistical analysis of the effect of various components of the subjects' facial appearance (third column) and the behaviour elicited in their interaction partners in ten decision situations (first column). Facial appearance is quantified by the face judgement scores regarding eight attributes (attractive, happy, generous, trustworthy, rational, risk-taking, greedy, angry) and the scores for their principal components (PC1, PC2; see Fig. 3a). The decision situations refer to the Dictator Game (DG: generosity), the Ultimatum Game (UG1: fairness, UG2: demand), the Trust Game (TG1: trust, TG2: trustworthiness), the Prisoner's Dilemma (PD: no free-riding), the Coordination Game (CG: coordination), the Snowdrift Game (SD: work share), the Stag Hunt Game (SH: risk-taking), and the Punishment Game (PG: punishment). The table shows the outcome of 100 linear regression analyses with a single predictor (each of the ten facial aspects) and a single dependent variable (the behaviour elicited in the interaction partners in the ten decision situations). In the case of [a] continuously scaled decisions, the dependent variable was quantified by the average amount of points received from the interaction partners; in the case of [b] binary scaled decisions, it was quantified by the average number of prosocial decisions received from the interaction partners. B – regression coefficient, R^2 – coefficient of determination, p – statistical significance. Significant associations are in **bold**.
